# Supplementary material for: In situ structure of actin remodeling during glucose-stimulated insulin secretion using cryo-electron tomography
Source: Nat Commun. 2024 Feb 12;15:1311. doi: 10.1038/s41467-024-45648-7 (PMC10861521; doi:10.1038/s41467-024-45648-7)
Supplement: Supplementary file 1 — Supplementary Information [file 41467_2024_45648_MOESM1_ESM.pdf]

Supplementary Information

In situ structure of actin remodeling during glucose-stimulated insulin secretion using cryo-electron tomography

Weimin Li[1,2], Angdi Li[1,2], Bing Yu[1,2], Xiaoxiao Zhang[1], Xiaoyan Liu[1], Kate L. White[3], Raymond C. Stevens[1,2], Wolfgang Baumeister[1,4,*], Andrej Sali[5,6,7,*], Marion Jasnin[8,*], Liping Sun[1,*]

[1] iHuman Institute, ShanghaiTech University, Shanghai 201210, China

[2] School of Life Science and Technology, ShanghaiTech University, Shanghai 201210, China

[3] Department of Chemistry, Bridge Institute, USC Michelson Center for Convergent Bioscience, University of Southern California, Los Angeles, CA 90089, USA

[4] Department of Molecular Structural Biology, Max Planck Institute of Biochemistry, 82152 Martinsried, Germany

[5] Quantitative Biosciences Institute, University of California, San Francisco, San Francisco, CA 94158, USA

[6] Department of Bioengineering and Therapeutic Sciences, University of California, San Francisco, San Francisco, CA 94158, USA

[7] Department of Pharmaceutical Chemistry, University of California, San Francisco, San Francisco, CA 94158, USA

[8] Helmholtz Pioneer Campus, Helmholtz Munich, 85764 Neuherberg, Germany; Department of Chemistry, Technical University of Munich, 85748 Garching, Germany.

Corresponding authors:

Wolfgang Baumeister: baumeist@biochem.mpg.de
Andrej Sali: sali@salilab.org
Marion Jasnin: marion.jasnin@helmholtz-munich.de
Liping Sun: sunlp@shanghaitech.edu.cn

Supplementary Information includes 10 Supplementary Figures, 3 Supplementary Tables and 8 Supplementary Movies.

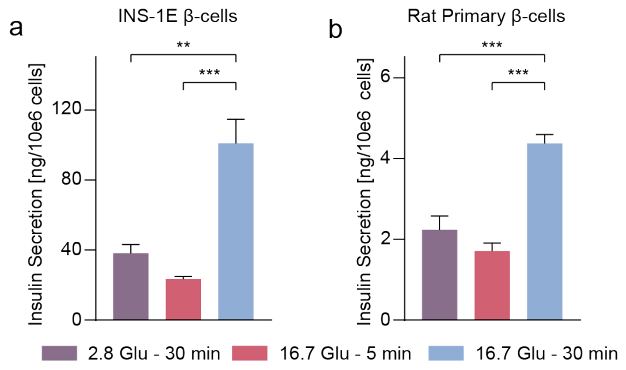

Supplementary Fig. 1 Insulin secretion levels under different conditions in both INS-1E β-cells and rat primary β-cells. Insulin secretion from INS-1E β-cells (**a**) and rat primary β-cells (**b**) was measured under different conditions by ELISA. For each condition, n = 4 biologically independent experiments. 2.8 Glu - 30 min versus 16.7 Glu - 30 min, **p = 0.0013; 16.7 Glu - 5 min versus 16.7 Glu - 30 min, ***p = 0.0003 in **a**. 2.8 Glu - 30 min versus 16.7 Glu - 30 min, ***p = 0.0007; 16.7 Glu - 5 min versus 16.7 Glu - 30 min, ***p = 0.0001 in **b**. *** indicates p<0.001. **** indicates p<0.0001 by one-way ANOVA. Data are presented as mean values +/- SEM. Source data are provided as a Source Data file.

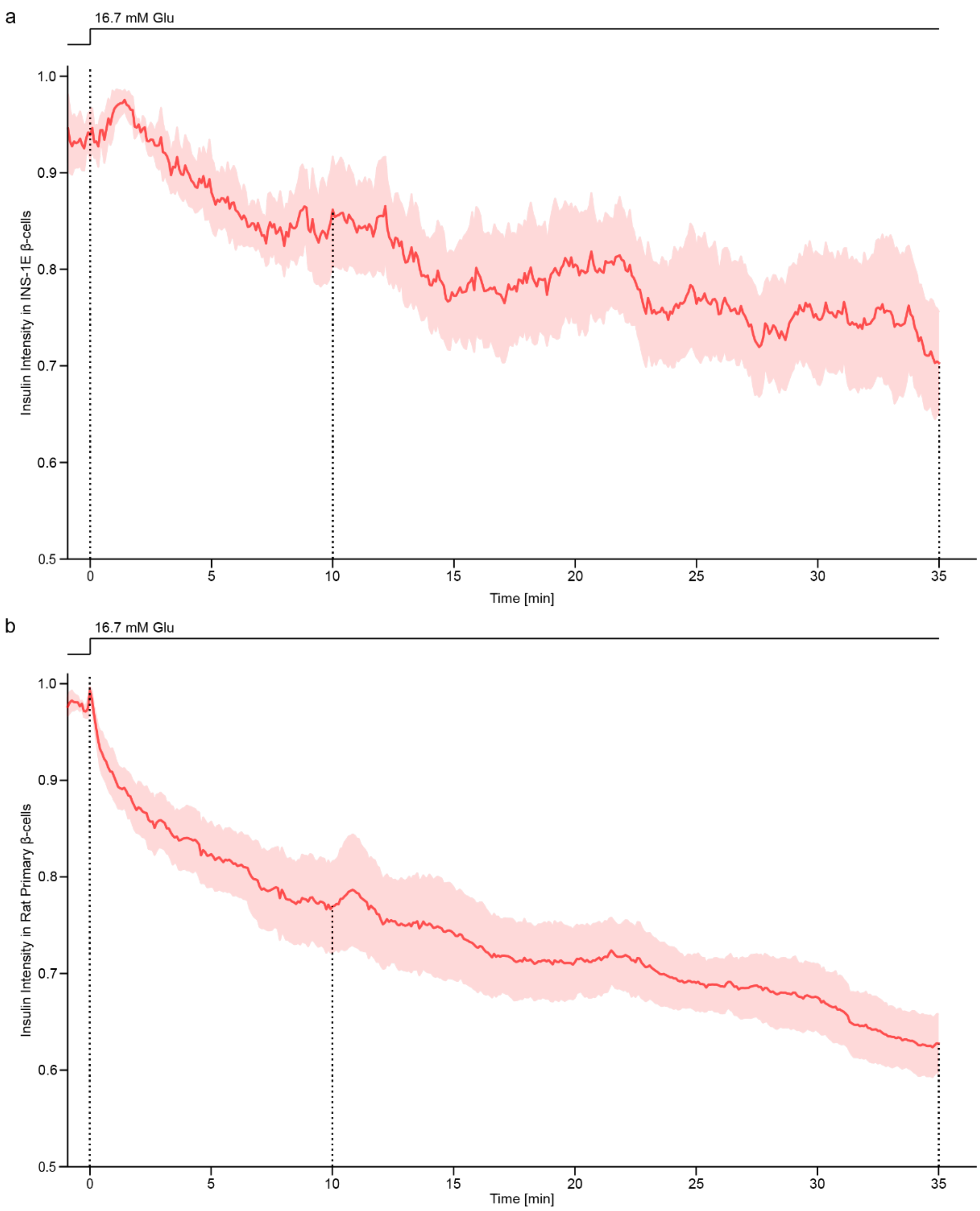

Supplementary Fig. 2 Quantification of insulin secretory granules in both INS-1E β-cells and rat primary β-cells. using live-cell total internal reflection fluorescence (TIRF) during GSIS. INS-1E β-cells (**a**) and rat primary β-cells (**b**) labeled with NPY for insulin secretory granules (red) were imaged by TIRF. Cells were starved in a 2.8 mM glucose KREB solution for 30 min, and then stimulated in a 16.7 mM glucose solution at 0 min. The fluorescence intensity of insulin secretory granules was recorded over time in live cells. A total of four TIRF images were collected from individual INS-1E β-cells and rat primary β-cells, respectively, all of which originated from biologically independent experiments. Signal intensities of insulin secretory granules were normalized by the maximum of the averaged intensity over four images. Data are presented as mean values +/- SEM (shaded area). Source data are provided as a Source Data file.

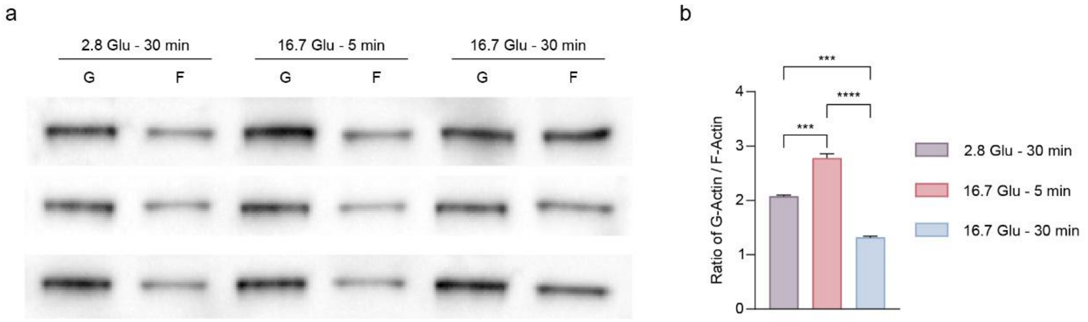

Supplementary Fig. 3 Quantitation of the globular/filamentous actin (G-actin / F-actin) ratio in INS-1E β-cells during GSIS. a Representative immunoblots of G-actin and F-actin. **b** G-actin / F-actin ratio under three conditions. For each condition, n = 3 biologically independent experiments. 2.8 Glu - 30 min versus 16.7 Glu - 5 min, ***p = 0.0002; 2.8 Glu - 30 min versus 16.7 Glu - 30 min, ***p = 0.0001; 16.7 Glu - 5 min versus 16.7 Glu - 30 min, ****p < 0.0001. * indicates p<0.05. ** indicates p<0.01. *** indicates p<0.001. **** indicates p<0.0001 by one-way ANOVA. Data are presented as mean values +/- SEM. Source data are provided as a Source Data file.

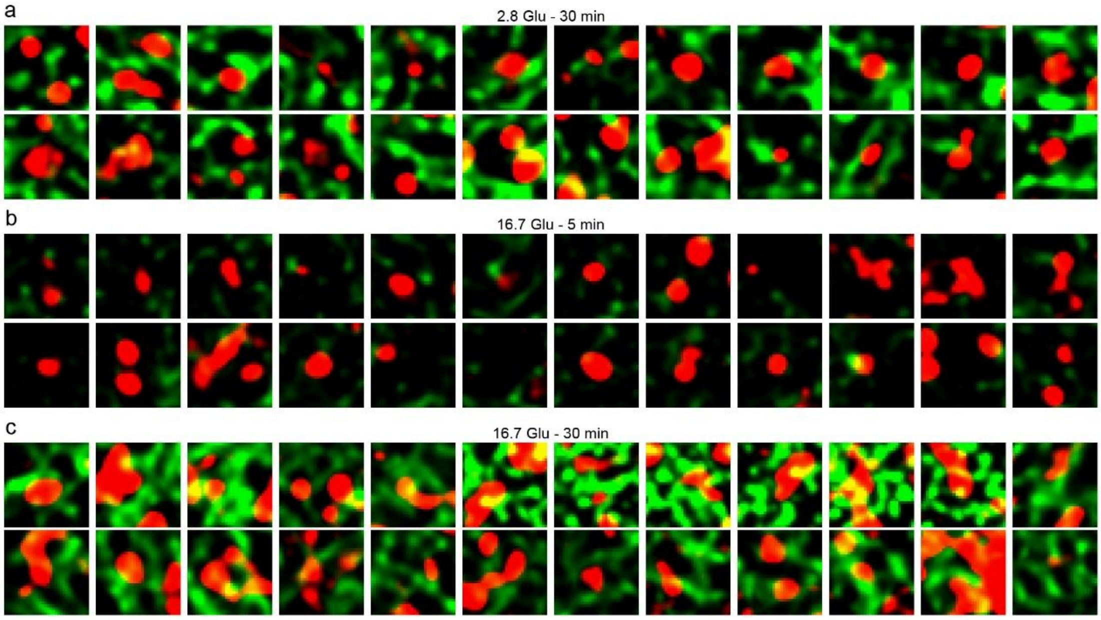

Supplementary Fig. 4 Subsections of INS-1E β-cells using SIM imaging during GSIS. INS-1E β-cells labeled with NPY for insulin secretory granules (red) and LifeAct for actin (green) were imaged using SIM. Cells were starved in a 2.8 mM glucose KREB solution for 30 min (**a**), and then stimulated in a 16.7 mM glucose solution for 5 min (**b**) and 30 min (**c**).

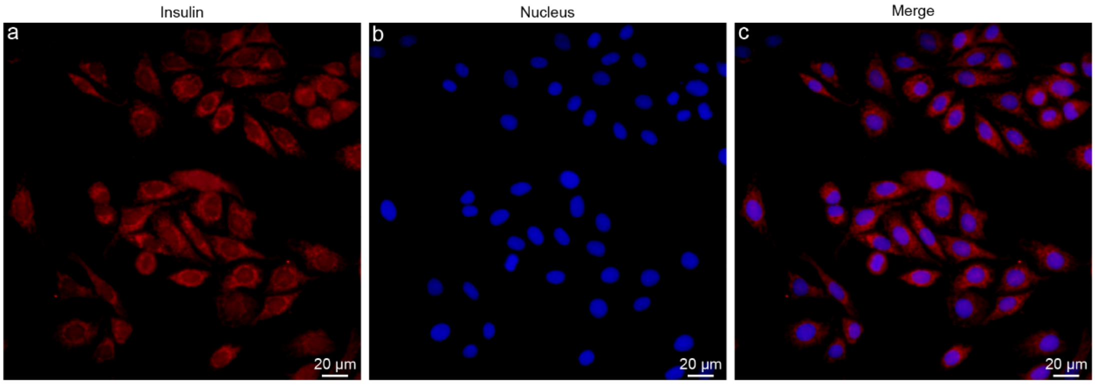

Supplementary Fig. 5 Validation of rat primary β-cells using immunofluorescence microscopy. Rat primary β-cells labeled with Cy3 for insulin secretory granules (red, **a**) and DAPI for the nucleus (blue, **b**) were imaged using widefield. These two channels were merged in **c**.

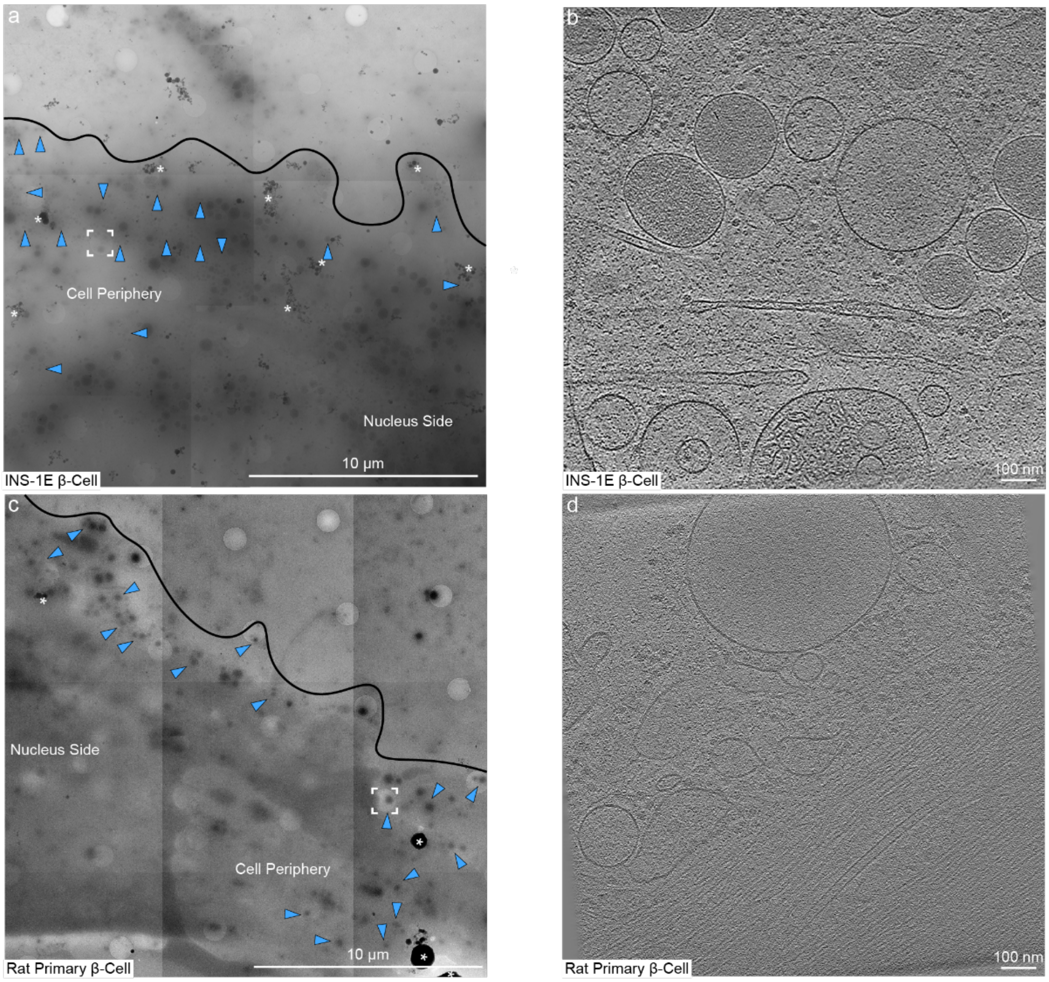

Supplementary Fig. 6 Low magnification view of the periphery of vitrified INS-1E β-cells and rat primary β-cells. An INS-1E β-cell (**a**) and a rat primary β-cell (**c**) vitrified on EM grids and imaged by cryo-TEM at 3600x and 4800x magnification, respectively. The edge of the cell is delineated on the map with a black line. The cell periphery (0-6 µm from the plasma membrane) is indicated on the map. Slices (**b** and **d**) through tomograms collected at the positions indicated by the white boxes in (**a**) and (**c**), respectively. Insulin secretory granules were labeled with blue arrows and ice labeled with white stars.

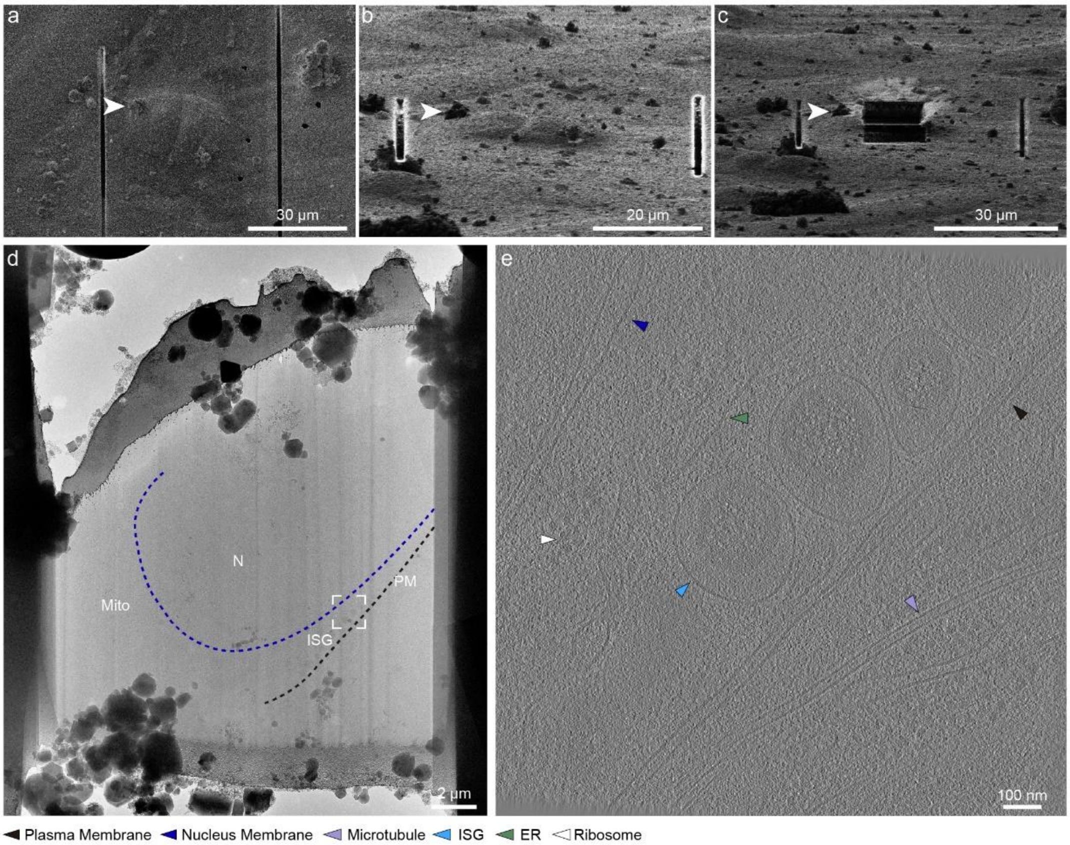

Supplementary Fig. 7 Cryo-FIB sample preparation of INS-1E β-cells for cryo-ET imaging. a Scanning electron microscope (SEM) image of frozen-hydrated INS-1E β-cells on an EM grid before FIB milling. **b, c** Ion Beam (IB) views of a 150-nm-thick lamella prepared in a plunge-frozen INS-1E cell before (**b**) and after (**c**) FIB milling. **d** Low magnification TEM overview of an INS-1E cell lamella. N, nucleus; Mito, mitochondrion; PM, plasma membrane; ISG, insulin secretory granule. **e** A 250-nm thick slice through a tomogram acquired at the position indicated by the white box in **d**. n =18 lamellas, corresponding to six tomograms for each condition from three biologically independent experiments.

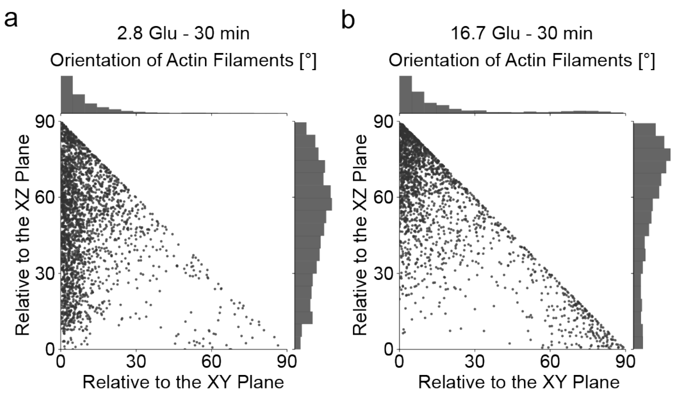

Supplementary Fig. 8 Quantitative analysis of actin filament orientations of the cell periphery data relative to the XY and XZ planes under 2.8 Glu - 30 min and 16.7 Glu - 30 min conditions. a-b Density map of the orientation of actin filaments relative to the XZ plane as a function of their orientation relative to the XY plane under 2.8 Glu - 30 min (**a**) and 16.7 Glu - 30 min (**b**) conditions. Each point on the density map reflects the corresponding density values by calculating kernel density. Source data are provided as a Source Data file.

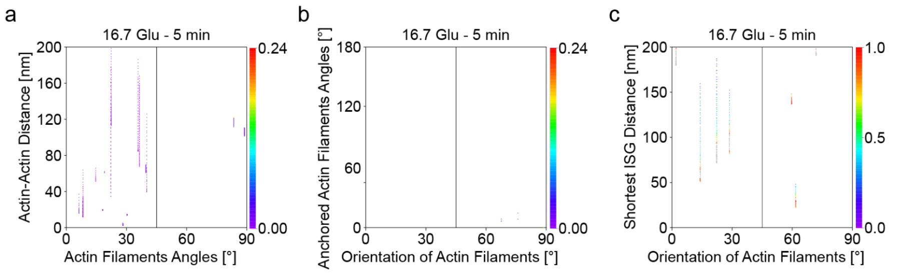

Supplementary Fig. 9 Quantitative analysis of actin filament organization at the cell periphery under the 16.7 Glu - 5 min condition. a Density map of the distance between actin filaments as a function of the angle between actin filaments during the 16.7 Glu – 5 min. **b** Density map of the distance between VM-anchored actin filaments as a function of their orientation relative to the VM during the 16.7 Glu – 5 min. **c** Density maps of the shortest distance between actin filaments and ISGs as a function of their orientation relative to the VM during the 16.7 Glu – 5 min. The color bar values range used in each analysis is consistent with the corresponding analyses conducted in the 2.8 Glu - 30 min and 16.7 Glu - 30 min conditions. Each point on the density map reflects the corresponding density values, calculated using kernel density. Source data are provided as a Source Data file.

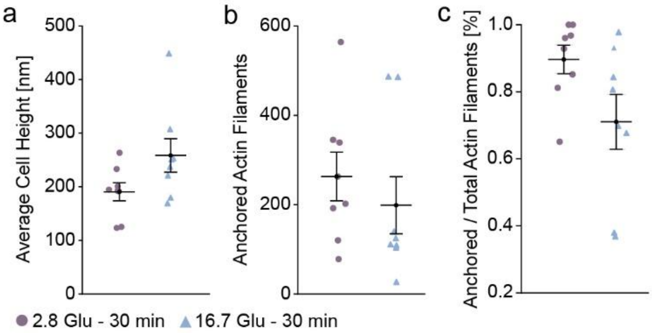

Supplementary Fig. 10 Cell height and the number of anchored actin filaments at the cell periphery. **a** Average height of the cell in each tomogram under different stimulation conditions. **b** Number of anchored actin filaments in each tomogram under different conditions. **c** Ratio of anchored filaments to total actin filaments under different conditions. Data are presented as mean values +/- SEM. Source data are provided as a Source Data file.

Supplementary Table 1 Number of SIM images under each condition

Cell type	Treatment	2.8 Glu - 30 min	16.7 Glu - 5 min	16.7 Glu - 30 min
INS-1E β-cell	No Y15	Six	Six	Six
	With Y15	Ten	Ten	Ten

Supplementary Table 2 Number of cryo-electron tomograms under each condition

Cell type	Position	2.8 Glu - 30 min	16.7 Glu - 5 min	16.7 Glu - 30 min
INS-1E β-cell	Cell periphery	Eight tomograms (four cells from two grids using Titan Krios G3)	Eight tomograms (six cells from three grids using Krios G4 and one cell from one grid using Titan Krios G3)	Eight tomograms (five cells from three grids using Titan Krios G3)
	Cell interior	Six tomograms (six cells from two grids using Krios G4)	Six tomograms (six cells from two grids using Krios G4)	Six tomograms (six cells from two grids using Krios G4)
Rat primary β-cell	Cell periphery	Four tomograms (three cells from two grids using Krios G4)	-	Four tomograms (three cells from two grids using Krios G4)

Supplementary Table 3 Cryo-ET data acquisition parameters

	Titan Krios G3	Krios G4
Magnification	26000	42000
Voltage (KV)	300	
Electron exposure (e-/A2)	~110-130	
Detector	Gatan K3	Falcon 4
Energy filter	Yes	Yes
Slit width	20	20
Phase plate	No	No
Defocus range (μm)	-5 to -7	-3 to -4, -5 to -7
Tilt range (min/max, starting angle, step)	-60° to +60°, 0°, 2° (cell periphery)	-60° to +60°, 0°, 2° (cell periphery); -70° to +50°, -10°, 2° (cell interior)
Tilt scheme	Dose-symmetric	
Pixel size (Å)	3.353	3.028
Tomograms (no.)	Seventeen INS-1E β-cell tomograms	Twenty five INS-1E β-cell tomograms; eight rat primary β-cell tomograms
Cell (no.)	Ten INS-1E β-cells	Twenty four INS-1E β-cells; six rat primary β-cells
Tilt axis	Y-axis	X-axis
