# Supplementary material for: In situ structure of actin remodeling during glucose-stimulated insulin secretion using cryo-electron tomography
Source: Nat Commun. 2024 Feb 12;15:1311. doi: 10.1038/s41467-024-45648-7 (PMC10861521; doi:10.1038/s41467-024-45648-7)
Supplement: Supplementary file 3 — Description of Additional Supplementary Files [file 41467_2024_45648_MOESM3_ESM.pdf]

# Description of Additional Supplementary Files

**File name: Supplementary Movie 1**

**Description: Biphasic insulin secretion during glucose-stimulated insulin secretion (GSIS) in INS-1E β-cells using total internal reflection fluorescence (TIRF) microscopy**. The green and red fluorescence represent actin filaments and insulin secretory granules, respectively. TIRF microscopy was employed to image in the vicinity of the ventral membrane (VM). TIRF imaging thickness is approximately 120 nm. High glucose stimulation was started at 55 seconds, and samples were collected over a total of 40 minutes. Scale bar: 5 μm.

**File name: Supplementary Movie 2**

**Description: Biphasic insulin secretion during GSIS in rat primary β-cells using TIRF microscopy**. The green and red fluorescence represent actin filaments and insulin secretory granules, respectively. TIRF microscopy was employed to image the VM. TIRF imaging thickness is approximately 120 nm. High glucose stimulation was started at 55 seconds, and samples were collected over a total of 40 minutes. Scale bar: 5 μm.

**File name: Supplementary Movie 3**

**Description: Tomogram and associated segmentation obtained at the cell periphery of an INS-1E β-cell under 2.8 Glu - 30 min condition.** Each organelle is labeled with a unique color, including actin filaments (orange), microtubules (violet), insulin secretory granules (blue), endoplasmic reticulum (ER; green), lysosomes (silver), and ribosomes (white).

**File name: Supplementary Movie 4**

**Description: Tomogram and associated segmentation obtained at the cell periphery of an INS-1E β-cell under 16.7 Glu - 5 min condition.** Each organelle is labeled with a unique color, including actin filaments (orange), microtubules (violet), insulin secretory granules (blue), ER (green), lysosomes (silver), and ribosomes (white).

**File name: Supplementary Movie 5**

**Description: Tomogram and associated segmentation obtained at the cell periphery of an INS-1E β-cell under 16.7 Glu - 30 min condition.** Each organelle is labeled with a unique color, including actin filaments (orange), microtubules (violet), insulin secretory granules (blue), ER (green), lysosomes (silver), and ribosomes (white).

**File name: Supplementary Movie 6**

**Description: Tomogram and associated segmentation obtained in the cell interior of an INS-1E β-cell under 2.8 Glu - 30 min condition.** Each organelle is labeled with a unique color, including actin filaments (orange), MTs (violet), ISGs (blue), ER (green), mitochondria (red), lysosomes (silver), and ribosomes (white).

**File name: Supplementary Movie 7**

**Description: Tomogram and associated segmentation obtained in the cell interior of an INS-1E β-cell under 16.7 Glu - 5 min condition.** Each organelle is labeled with a unique color, including actin filaments (orange), MTs (violet), ISGs (blue), ER (green), mitochondria (red), lysosomes (silver), and ribosomes (white).

**File name: Supplementary Movie 8**

**Description: Tomogram and associated segmentation obtained in the cell interior of an INS-1E β-cell under 16.7 Glu - 30 min condition.** Each organelle is labeled with a unique color, including actin filaments (orange), MTs (violet), ISGs (blue), ER (green), mitochondria (red), lysosomes (silver), and ribosomes (white).
