# Supplementary material for: In situ structure of actin remodeling during glucose-stimulated insulin secretion using cryo-electron tomography
Source: Nat Commun. 2024 Feb 12;15:1311. doi: 10.1038/s41467-024-45648-7 (PMC10861521; doi:10.1038/s41467-024-45648-7)
Supplement: Supplementary file 12 — Reporting Summary [file 41467_2024_45648_MOESM12_ESM.pdf]

Corresponding author(s): Wolfgang Baumeister, Andrej Sali, Marion Jasnin, Liping Sun

Last updated by author(s): Jan 11, 2024

Reporting Summary

Nature Portfolio wishes to improve the reproducibility of the work that we publish. This form provides structure for consistency and transparency in reporting. For further information on Nature Portfolio policies, see our Editorial Policies and the Editorial Policy Checklist.

Statistics

For all statistical analyses, confirm that the following items are present in the figure legend, table legend, main text, or Methods section.

n/a	Confirmed	
☐	☒	The exact sample size (*n*) for each experimental group/condition, given as a discrete number and unit of measurement
☐	☒	A statement on whether measurements were taken from distinct samples or whether the same sample was measured repeatedly
☐	☒	The statistical test(s) used AND whether they are one- or two-sided<br>*Only common tests should be described solely by name; describe more complex techniques in the Methods section.*
☐	☒	A description of all covariates tested
☐	☒	A description of any assumptions or corrections, such as tests of normality and adjustment for multiple comparisons
☐	☒	A full description of the statistical parameters including central tendency (e.g. means) or other basic estimates (e.g. regression coefficient) AND variation (e.g. standard deviation) or associated estimates of uncertainty (e.g. confidence intervals)
☐	☒	For null hypothesis testing, the test statistic (e.g. *F*, *t*, *r*) with confidence intervals, effect sizes, degrees of freedom and *P* value noted<br>*Give P values as exact values whenever suitable.*
☒	☐	For Bayesian analysis, information on the choice of priors and Markov chain Monte Carlo settings
☒	☐	For hierarchical and complex designs, identification of the appropriate level for tests and full reporting of outcomes
☒	☐	Estimates of effect sizes (e.g. Cohen's *d*, Pearson's *r*), indicating how they were calculated

Our web collection on statistics for biologists contains articles on many of the points above.

Software and code

Policy information about availability of computer code

Data collection	Insulin secretion data was collected by SoftMax Pro (v5.4.5.000, Molecular Devices).Total internal reflection fluorescence (TIRF) data was collected by NIS-Elements AR 64-bit (v5.21.00, Nikon); Western blot data was collected by Image Lab (v5.2, Bio-Rad); Structured illumination microscopy (SIM) data was collected by Zen 64-bit (3.0 SR FP1 black, Carl Zeiss); Focused ion beam (FIB) data was collected by Microscope Control (v20.1.1, Thermo Fisher Scientific); Tomography data was collected by TEM User Interface (v2.15.3, Thermo Fisher Scientific) with SerialEM (v3.8.0 beta, University of Colorado) and TEM User Interface (v3.9.1, Thermo Fisher Scientific) with Tomography (v5.8.0.3166REL, Thermo Fisher Scientific).
Data analysis	Insulin secretion data was analyzed by GraphPad Prism (v9.4.1, GraphPad Software). Total internal reflection fluorescence (TIRF) data was analyzed by Huygens (v19.4, Scientific Volume Imaging), Image J (v1.53f51) and homemade Python scripts (v3.7.7); Western blot were analyzed by Image J (v1.53f51) and GraphPad Prism (v9.4.1, GraphPad Software); Structured illumination microscopy (SIM) was analyzed by Image J (v1.53f51) and GraphPad Prism (v9.4.1, GraphPad Software); Tomography data was analyzed by Tomoman (https://github.com/williamnwan/TOMOMAN), MATLAB (R2019b, MathWorks), MotionCorr2 (v1.5.0, UCSF), IMOD (v4.9.12, University of Colorado), CryoCare (v0.2.2, https://github.com/juglab/cryoCARE_T2T), Amira (v2019.2, Thermo Fisher Scientific), GraphPad Prism (v9.4.1, GraphPad Software), OriginPro 2021 (9.8.0.200) and homemade Python scripts (v3.7.7). Specifically, Python scripts used for actin filament and microtubule resample, Actin-Actin distance, actin filaments angles, anchored actin filament angles, Actin-ISG distance, MT-ISG distance, and shortest MT distance of actin filament are available at https://github.com/SaliLab-SH/iPA/tree/ActinAnalysis.

For manuscripts utilizing custom algorithms or software that are central to the research but not yet described in published literature, software must be made available to editors and reviewers. We strongly encourage code deposition in a community repository (e.g. GitHub). See the Nature Portfolio guidelines for submitting code & software for further information.

Data

Policy information about availability of data

All manuscripts must include a data availability statement. This statement should provide the following information, where applicable:

- Accession codes, unique identifiers, or web links for publicly available datasets
- A description of any restrictions on data availability
- For clinical datasets or third party data, please ensure that the statement adheres to our policy

The data generated in this study are provided in the Supplementary Information/Source Data file. Source data are provided with this paper. The tomograms used in this study are available in Electron Microscopy Data Bank (EMDB) under accession codes: EMD-35841, EMD-35809, EMD-35842, EMD-35843, EMD-35844, EMD-35845, EMD-35846, EMD-35847, EMD-35855, EMD-35840, EMD-35856, EMD-35857, EMD-35858, EMD-35859, EMD-35860, EMD-35861, EMD-35849, EMD-35850, EMD-35851, EMD-35852, EMD-35839, EMD-35848, EMD-35853, EMD-35854, EMD-35935, EMD-35874, EMD-35896, EMD-35875, EMD-35876, EMD-35885, EMD-35886, EMD-35887, EMD-35897, EMD-35889, EMD-35890, EMD-35936, EMD-35891, EMD-35892, EMD-35893, EMD-35894, EMD-35937, EMD-35895, EMD-37278, EMD-37279, EMD-37280, EMD-37281, EMD-37282, EMD-37283, EMD-37284, EMD-37285.

Research involving human participants, their data, or biological material

Policy information about studies with human participants or human data. See also policy information about sex, gender (identity/presentation), and sexual orientation and race, ethnicity and racism.

Reporting on sex and gender	N/A
Reporting on race, ethnicity, or other socially relevant groupings	N/A
Population characteristics	N/A
Recruitment	N/A
Ethics oversight	N/A

Note that full information on the approval of the study protocol must also be provided in the manuscript.

Field-specific reporting

Please select the one below that is the best fit for your research. If you are not sure, read the appropriate sections before making your selection.

☒ Life sciences ☐ Behavioural & social sciences ☐ Ecological, evolutionary & environmental sciences

For a reference copy of the document with all sections, see nature.com/documents/nr-reporting-summary-flat.pdf

Life sciences study design

All studies must disclose on these points even when the disclosure is negative.

Sample size	The sample size (4-8 tomograms from 3-6 cells in each condition) was chosen because of the practical limitations of the methodology used. It was sufficient to interpret the data in a robust manner according to previous publications (see for example Figure4 in Trinkaus, V.A., Riera-Tur, I., Martínez-Sánchez, A. et al. Nat Commun 12, 2110 (2021)).
Data exclusions	No data were excluded from this study.
Replication	All experiments have been conducted for at least three times with similar results.
Randomization	Randomization was not necessary for this basic science study, which did not include clinical trials. All samples used in each set of experiments were equal.
Blinding	Blinding is not relevant to this study because it is not a comparative study.

Reporting for specific materials, systems and methods

We require information from authors about some types of materials, experimental systems and methods used in many studies. Here, indicate whether each material, system or method listed is relevant to your study. If you are not sure if a list item applies to your research, read the appropriate section before selecting a response.

Materials & experimental systems

n/a	Involved in the study	
☐	☒	Antibodies
☐	☒	Eukaryotic cell lines
☒	☐	Palaeontology and archaeology
☒	☐	Animals and other organisms
☒	☐	Clinical data
☒	☐	Dual use research of concern
☒	☐	Plants

Methods

n/a	Involved in the study	
☒	☐	ChIP-seq
☒	☐	Flow cytometry
☒	☐	MRI-based neuroimaging

Antibodies

Antibodies used

1. Insulin primary antibody (Affinity, AF5109).
2. Goat anti-mouse IgG (Proteintech, SA00001-1).
3. Actin primary antibody in G-Actin/F-Actin In Vivo Assay Biochem Kit (Cytoskeleton, BK037).
4. Goat Anti-Rabbit IgG (Abcam, ab6721).

Validation

All antibodies used have been validated by the manufacturer. Please find websites at below:
1. https://www.affbiotech.com/goods-4416-AF5109-Insulin_Antibody.html
2. https://www.ptgcn.com/products/HRP-conjugated-Affinipure-Goat-Anti-Mouse-IgG-H-L-secondary-antibody.htm
3. https://www.cytoskeleton.com/bk037
4. https://www.abcam.com/products/secondary-antibodies/goat-rabbit-igg-hl-hrp-ab6721.html

Eukaryotic cell lines

Policy information about cell lines and Sex and Gender in Research

Cell line source(s)

Clonal rat INS-1E β-cells was gifted from P. Maechler's laboratory at the University of Geneva.
Rat primary β-cells was bought from Shanghai Zhong Qiao Xin Zhou Biotechnology Co.,Ltd.

Authentication

Clonal rat INS-1E β-cells and rat primary β-cells were authenticated based on their morphology, growth condition, and insulin secretion level.

Mycoplasma contamination

Clonal rat INS-1E β-cells and rat primary β-cells were tested with a PCR based kit (40612ES60, Yeasen), and no mycoplasma contamination was found.

Commonly misidentified lines
(See ICLAC register)

No commonly misidentified cell lines were used.

Plants

Seed stocks

N/A

Novel plant genotypes

N/A

Authentication

N/A
